# Supplementary material for: Species variations in tenocytes’ response to inflammation require careful selection of animal models for tendon research
Source: Sci Rep. 2021 Jun 14;11:12451. doi: 10.1038/s41598-021-91914-9 (PMC8203623; doi:10.1038/s41598-021-91914-9)
Supplement: Supplementary file 1 — Supplementary Files. [file 41598_2021_91914_MOESM1_ESM.docx]

# Species variations in tenocytes’ response to inflammation require careful selection of animal models for tendon research

# Author list: Gil Oreff^1+^, Michele Fenu^1+^, Claus Vogl^2^, Iris Ribitsch^1^, Florien Jenner^1*^

1 University of Veterinary Medicine Vienna, Department of Companion Animals and Horses, Equine Surgery Unit, VETERM, Veterinaerplatz 1, 1210 Vienna, Austria

2 University of Veterinary Medicine Vienna, Department of Biomedical Sciences, Institute of Animal Breeding and Genetics, Veterinaerplatz 1, 1210 Vienna, Austria

# Supplementary Materials

##
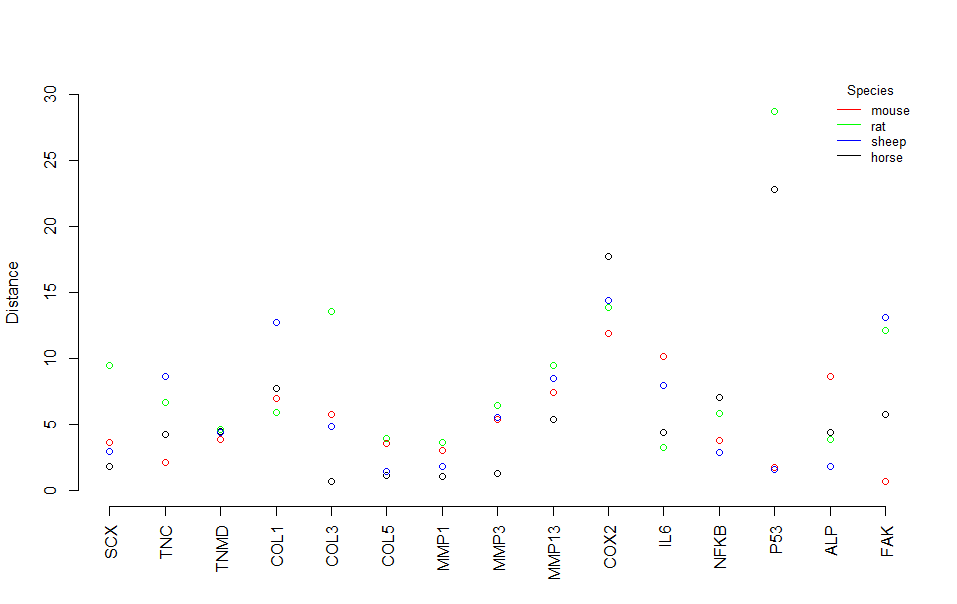


Supplementary figure 1: Graph of the Mahalonobis distances of the four model species to human for each gene


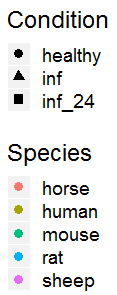

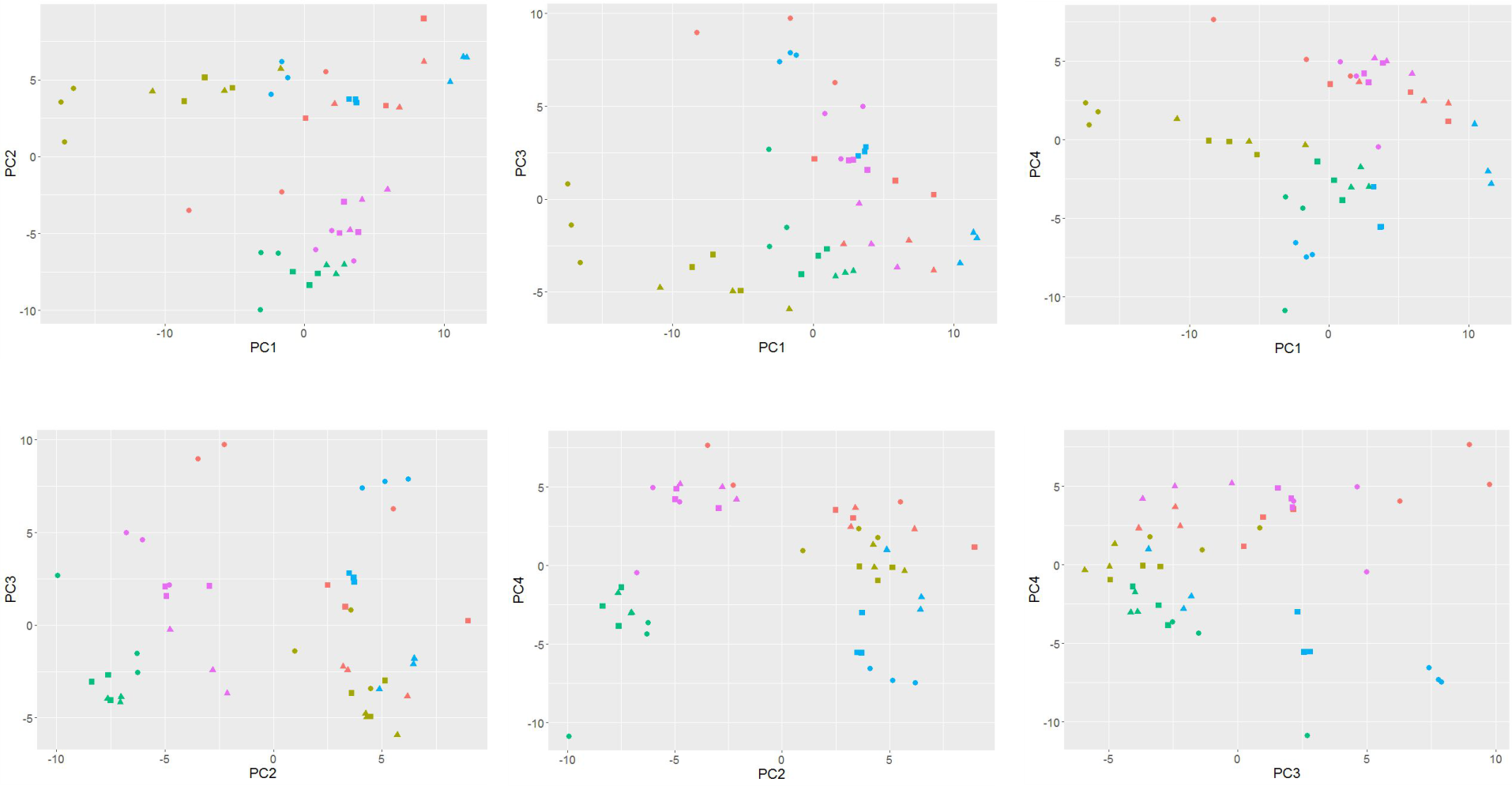


Supplementary figure 2: Pairwise plots of a Principal Component Analysis of gene expression values with PC1 explaining 36% of the variance, PC2 23%, PC3 14% and PC4 13%. Species are colour coded, conditions (healthy, transiently inflamed and continuously inflamed) are differentiated by symbols.

Supplementary table 1: The qPCR results (log2 FC relative to GAPDH) for each of the 15 genes (3 biological replicates/species) are listed for each condition.

Supplementary table 2: qPCR Primer sequences
